# Genomic Analysis of Doxycycline Resistance–Associated 16S rRNA Mutations in *Treponema pallidum* Subspecies *pallidum*

**DOI:** 10.3201/eid3202.251060

**Published:** 2026-02

**Authors:** George S. Long, Mackenzie Neale, Thomas Braukmann, Vanessa Tran, Nishant Singh, Vanessa Allen, Muhammad Morshed, Jessica Minion, Paul Van Caeseele, Maud Vallée, Todd Hatchette, Samir N. Patel, Raymond S.W. Tsang, Venkata R. Duvvuri

**Affiliations:** Public Health Ontario, Toronto, Ontario, Canada (G.S. Long, T. Braukmann, V. Tran, N. Singh, V. Allen, S.N. Patel, V.R. Duvvuri); Public Health Agency of Canada, Winnipeg, Manitoba, Canada (M. Neale, R.S.W. Tsang); University of Toronto, Toronto (T. Braukmann, V. Tran, V. Allen, S.N. Patel, V.R. Duvvuri); Sinai Health and University Health Network, Toronto (V. Allen); British Columbia Centre for Disease Control, Vancouver, British Columbia, Canada (M. Morshed); University of British Columbia, Vancouver (M. Morshed); Roy Romanow Provincial Laboratory, Regina, Saskatchewan, Canada (J. Minion); University of Saskatchewan, Regina (J. Minion); Cadham Provincial Laboratory, Winnipeg (P. Van Caeseele); University of Manitoba, Winnipeg (P. Van Caeseele); Laboratoire de Santé Publique du Québec, Sainte-Anne-de-Bellevue, Quebec, Canada (M. Vallée); Dalhousie University, Halifax, Nova Scotia, Canada (T. Hatchette); York University, Toronto (V.R. Duvvuri)

**Keywords:** *Treponema pallidum*, sexually transmitted infections, bacteria, antimicrobial resistance, doxycycline, 16S rRNA, comparative genomics, genomic variants, Canada

## Abstract

We inspected 16S rRNA sequences of 784 publicly available *Treponema pallidum* subspecies *pallidum* genomes and 17 new *T. pallidum* subsp. *pallidum* genomes from Canada for putative mutations associated with doxycycline resistance. These findings establish a global genomic baseline for monitoring doxycycline resistance in syphilis.

*Treponema pallidum* subspecies *pallidum* (TPA) is the causative agent of syphilis, a sexually transmitted infection (STI) that is on the rise globally. The incidence rate in Canada has increased by 77% since 2018, reaching 30.5 cases of syphilis/100,000 persons as of 2023 (*1*). Although syphilis is traditionally treated with benzathine penicillin G, recent shortages have hampered treatment (*2*). In Canada, doxycycline is the recommended alternative treatment for primary, secondary, and early latent syphilis in nonpregnant adults who are allergic to penicillin. Doxycycline is also an effective preexposure and postexposure prophylactic for bacterial STIs (*3*); however, treatment failures have been reported in cases of secondary and early latent syphilis (*4*).

Concerns have been raised about the mass use of doxycycline as a prophylactic in at-risk communities (*3*) despite its demonstrated effectiveness. The primary concern is the potential selection for doxycycline-resistant sexually transmitted bacteria and alterations to the gut microflora (*3*). Those worries stem from the fact that some doxycycline resistance mechanisms, such as Tet efflux pumps (*5*), are horizontally transferred through plasmids or transposons and thus render future treatments for unrelated infections less effective. Other routes to resistance, such as ribosomal mutations to the 16S rRNA gene (*5*–*8*), are possible. In *T. pallidum*, doxycycline resistance is hypothesized to occur through mutations in the 16S rRNA genes because the pathogen is believed to rarely undergo recombination (*9*,*10*). In light of that factor, a previous study demonstrated that repeated exposure to doxycycline did not significantly increase resistance (*11*). A recent genomic analysis (*12*) identified a mutational triplet at positions 965–967 (*E. coli* numbering) in the 16S rRNA gene of *Treponema* and *Spirochaeta*, which might warrant continued surveillance and further investigation for their potential role in tetracycline resistance.

The goal of this study was to characterize 17 newly sequenced TPA genomes from Canada and monitor antimicrobial resistance (AMR), with a focus on doxycycline. This analysis also includes 784 previously published global TPA genomes and sequencing libraries from the National Center for Biotechnology Information (NCBI) GenBank and Sequence Read Archives (Appendix). We investigated mutations associated with tetracycline-resistant *Cutibacterium acnes*, *Escherichia coli*, and *Helicobacter pylori* (*6*–*8*) and translated those resistance-associated positions to the 16S rRNA coordinate system of TPA to support future research and public health genomic surveillance. We also investigated macrolide resistance by analyzing known mutations in the 23S rRNA gene (*13*). This project received ethics review clearance from Health Canada and Public Health Agency of Canada’s Research Ethics Borad (file no. REB 2023-012P).

## The Study

We conducted genome enrichment of the study genomes by using Agilent SureSelect protocol (https://www.agilent.com) before sequencing. Lineage classification using both the 3-gene multilocus sequence typing (MLST) scheme (*14*) and the core genome lineages generated with PopPUNK (*15*) confirmed that the strains were part of the TPA subspecies. Most (15/17) of the TPA genomes belonged to the SS14 lineage; the remaining genomes belonged to the Nichols lineage (Table 1; Appendix Figures 1–4). Core genome–based classification outperformed the MLST because it successfully identified the lineages of all 17 samples, whereas MLST failed for 2 samples. 

**Table 1:** Demographic and genomic features of 17 *Treponema pallidum* samples from adults in Canada collected during 2016–2024 used in genomic analysis of doxycycline resistance–associated 16S rRNA mutations in *T. pallidum* subsp. pallidum*

Sample	GenBank or BioSample accession no.	Year	Province	Lineage		Macrolide resistant
				MLST	PopPUNK	
MB1	CP191228	2016	Manitoba	SS14	SS14	Y
SK1	CP191227	2023	Saskatchewan	SS14	SS14	Y
CDN1	SAMN49899419	2016	Manitoba	SS14	SS14	Y
CDN2	SAMN49899420	2022	Manitoba	SS14	SS14	Y
CDN3	SAMN49899421	2024	British Columbia	SS14	SS14	Y
CDN4	SAMN49899422	2024	British Columbia	Unknown	SS14	Y
CDN5	SAMN49899423	2024	Saskatchewan	SS14	SS14	Y
CDN6	SAMN49899424	2024	Saskatchewan	SS14	SS14	Y
CDN7	SAMN49899425	2024	Saskatchewan	SS14	SS14	Y
CDN8	SAMN49899426	2024	Saskatchewan	SS14	SS14	N
CDN9	SAMN49899427	2024	Saskatchewan	Unknown	Nichols	Y
CDN10	SAMN49899428	2024	Saskatchewan	SS14	SS14	Y
CDN11	SAMN49899429	2024	Saskatchewan	SS14	SS14	Y
CDN12	SAMN49899430	2024	Québec	SS14	SS14	Y
CDN13	SAMN49899431	2024	Québec	SS14	SS14	Y
CDN14	SAMN49899432	2024	Saskatchewan	SS14	SS14	Y
CDN15	SAMN49899433	2024	Nova Scotia	Nichols	Nichols	Y

*The TPA lineages were identified with both MLST and PopPUNK (*14*,*15*) (Appendix). Macrolide resistance was identified on the basis of single-nucleotide polymorphisms in the 23S rRNA gene (*13*). Samples were obtained from adults (18–64 years of age), apart from 2 (BC1 and BC2) that did not contain any identifying information.

Most genomes from Canada were sampled in Saskatchewan in 2024 during a provincewide syphilis outbreak in the Prairies (Table 1). Those samples were geographically dispersed, likely suggesting multiple independent transmission events; however, detailed sexual contact tracing information is not available. We used the additional 784 TPA genomes from public databases (505 genomes from GenBank, 279 de novo assembled from data in previous studies [Appendix]) to determine the breadth of the mutational landscape of the 16S rRNA gene.

We retrieved reference sequences of 16S rRNA genes from *C. acnes* (NR_040847.1:1–1486), *E. coli* (U00096.1:4166659–8200), *H. pylori* (CP003904.1:1512657–1157), and TPA (Nichols lineage NC_021490.2:231255–23280 and SS14 lineage NC_021508.1:231297–2845) from NCBI and aligned them using MAFFT to translate known tetracycline resistance–associated mutation sites (*6*–*8*,*11*) (Table 2) to the TPA coordinate system. Specifically, the mutation at position 1032 in *C. acnes* corresponds to position 1062 in TPA; the *E. coli* mutation at position 964 aligns to position 967 and the *E. coli* mutation at position 1053–1055 aligns to positions 1057–1059 in TPA. Similarly, the *H. pylori* resistance–associated triplet at positions 965–967 (*6*,*11*) maps to 968–970 in TPA (Table 3). The coordinates of the reported mutational triplet in *T. pallidum* at positions 965–967 appear to be relative to *E. coli* (*11*), because the mutational triplet was identified at positions 968–970 in our reference strains. We manually inspected known tetracycline resistance–associated positions (Table 2) for mutations (Appendix).

**Table 2:** 16S rRNA mutations related to tetracycline resistance in genomic analysis of doxycycline resistance-associated 16S rRNA mutations in *Treponema pallidum* subsp. pallidum*

Species	Resistance mutations using *E. coli* (U00096.1) coordinates	Species-specific coordinates	Reference
*Escherichia coli *	A964G, G1053A, C1054T, A1055G	A966G, G1056A, C1057T, A1058G	(*8*)
*Cutibacterium acnes*	G1058C	G1032C	(*7*)
*Helicobacter pylori*	A965G, A965G & A967C, A965G & G966T, A967C	A930G, A930G & A932C, A930G & G931T, A932C	(*6*)

*Ampersands in the resistance mutations column indicate multi-allele resistance variants.

**Table 3:** Conversion of key tetracycline-related mutations to 16S coordinates in *Treponema pallidum *subsp.* pallidum* in genomic analysis of doxycycline resistance–associated 16S rRNA mutations in *T. pallidum* subsp. pallidum*

TPA	Position	965	966	967	968	969	970	972	973	1055	1056	1057	1058	1059	1060	1061	1063	1063
	Nichols	C	G	A	T	G	A	T	A	C	T	G	C	A	T	G	G	C
	SS14	C	G	A	T	G	A	T	A	C	T	G	C	A	T	G	G	C
*C. acnes*	Position	935	936	937	938	939	940	941	942	1025	1026	1027	1028	1029	1030	1031	1032	1033
	WT	C	G	A	T	G	C	A	A	G	T	G	C	A	T	G	G*	C
	Hypothetical AMR strain	C	G	A	T	G	C	A	A	G	T	G	C	A	T	G	c	C
*E. coli*	Position	962	963	964	965	966	967	968	969	1051	1052	1053	1054	1055	1056	1057	1058	1059
	WT	C	G	A*	T	G	C	A	A	C	T	G*	C*	A*	T	G	G	C
	Hypothetical AMR strain	C	G	g	T	G	C	A	A	C	T	a	t	g	T	G	G	C
*H. pylori*	Position	927	928	929	930	931	932	933	934	1021	1022	1023	1024	1025	1026	1027	1028	1029
	WT	C	G	A	A*	G*	A*	T	A	C	T	G	C	A	C	G	G	C
	Hypothetical AMR strain	C	G	A	g	t	c	T	A	C	T	G	C	A	C	G	G	C

*Alignment of 16S genes from *E. coli* (U00096.1, 4166659–4168200), *C. acnes* (NR_040847.1, 1–1486), *H. pylori* (CP003904.1, 1512657–1511157), and TPA (Nichols lineage NC_021490.2:231255–232803 and SS14 lineage NC_021508.1:231297–232845). Columns in gray indicate sites related to tetracycline resistance whereas cells with an asterisk reveal the reference bacteria and nucleotide. Lowercase letters indicate a mutation in comparison to the WT sequence. The final coordinates reported in this dispatch are for the TPA 16S rRNA gene. Flanking sites are shown to give additional context. AMR, antimicrobial resistant; TPA, *Treponema pallidum *subsp.* pallidum*; WT, wild-type.

Alignment of the 16S rRNA genes also revealed that the single-nucleotide polymorphisms (SNPs) associated with doxycycline resistance in *C. acnes* and *E. coli* share the same wild-type background as TPA (Table 2). Because a single mutation at those sites can potentially cause doxycycline resistance (*7*,*8*), similar mutations could possibly exert comparable effects in TPA. In contrast, resistance-associated mutations in *H. pylori* arise from a unique wild-type background relative to *C. acnes*, *E. coli*, and TPA. If the composition of the nucleotide triplet is key to conferring doxycycline resistance, then 3 of the 4 known resistance alleles will require 2 nucleotide substitutions in TPA. The only *H. pylori* resistance allele that requires a single mutation in TPA is gGA (T967G) (*6*). None of the 784 publicly available TPA genomes nor the 17 Canada TPA genomes contained mutations in the 16S rRNA gene.

We called the 16S rRNA variants using a diploid model for TPA to account for the presence of 2 gene copies. To confirm that those findings were not methodological artifacts, we analyzed the 23S rRNA gene and found it to be duplicated. Specifically, we investigated the A2058G and A2059G SNPs in *E. coli* (*13*) that correspond to positions 2106 and 2107 in TPA. We detected macrolide resistance in 94% (16/17) (Table 1) of the Canada genomes through a A2106G mutation and in ≈66% (514/784) of the publicly available TPA genomes, supporting the robustness of those AMR findings.

## Conclusions

The increasing use of doxycycline as a prophylactic for syphilis presents a growing risk for the emergence of AMR. Given the genetic stability of *T. pallidum* (*10*), genomic surveillance programs should prioritize monitoring positions 967–970, 1057–1059, and 1062 (TPA numbering) of the 16S rRNA genes, because those sites could serve as early indicators of emerging doxycycline resistance (*6*–*9*,*11*). Their relationship to doxycycline resistance in TPA remains theoretical and based on comparative genomics (*5*–*7*); in vitro phenotypic validation will be essential to determine their functional significance (*11*). Future analyses must account for the presence of 2 copies of the 16S rRNA gene in TPA, because heterozygous alleles could attenuate the phenotypic expression of doxycycline resistance. Nevertheless, our work establishes a global baseline for 16S rRNA diversity in TPA, simplifying future doxycycline resistance surveillance. 

Appendix Additional information about genomic analysis of doxycycline resistance-associated 16S rRNA mutations in *T. pallidum* subsp. *pallidum*
