# Correction: Vol. 32, No. 2

**DOI:** 10.3201/eid3208.C13208

**Published:** 2026-08

**Keywords:** errata, erratum, correction

Some details of the analyses were incorrect in Genomic Analysis of Doxycycline Resistance–Associated 16S rRNA Mutations in *Treponema pallidum* Subspecies *pallidum* (G.S. Long et al.). The article has been corrected online (https://wwwnc.cdc.gov/eid/article/32/2/25-1060_article"https://wwwnc.cdc.gov/eid/article/32/2/25-1060_article").

