# Supplementary material for: Genomic Analysis of Doxycycline Resistance–Associated 16S rRNA Mutations in Treponema pallidum Subspecies pallidum
Source: Emerg Infect Dis. 2026 Feb;32(2):242–5. doi: 10.3201/eid3202.251060 (PMC12928234; doi:10.3201/eid3202.251060)
Supplement: Appendix — Additional information about genomic analysis of doxycycline resistance-associated 16S rRNA mutations in T. pallidum subsp. pallidum [file 25-1060-Techapp-s1.pdf]

Article DOI: https://doi.org/10.3201/eid3202.251060

EID cannot ensure accessibility for supplementary materials supplied by authors. Readers who have difficulty accessing supplementary content should contact the authors for assistance.

Genomic Analysis of Doxycycline Resistance–Associated 16S rRNA Mutations in *Treponema pallidum* Subspecies *pallidum*

Appendix

This study analyzed 784 *T. pallidum* subsp. *pallidum* (TPA) genomes, comprising previously published global genomes from NCBI and SRA (n = 767), along with 17 newly sequenced Canadian genomes generated in this study. The following sections provide methodological approaches.

Sample Source, Sequencing, and Consensus Building

Clinical swab samples from across Canada were submitted to the National Microbiology Laboratory (NML) in Winnipeg, Manitoba for confirmatory TPA PCR testing by provincial public health agencies. These submissions represent routine clinical diagnostic requests rather than targeted sampling. The samples were screened for TPA DNA quantity, and those with *polA* gene concentrations >1,000 copies/µl were selected for sequencing to ensure adequate genome recovery and high-confidence variant calling. Table 1 presents 17 Canadian TPA genomes included samples collected between 2016 and 2024 from Canadian provinces: Manitoba, Saskatchewan, British Columbia, Quebec, and Nova Scotia. Eight of the seventeen Canadian genomes were sampled in Saskatchewan in 2024 during a province-wide syphilis outbreak in the Prairies. These cases were geographically dispersed across Saskatchewan. Although this distribution likely suggests multiple independent transmission events, detailed sexual contact tracing information is not available.

Sample extraction was performed using a QIAamp DNA Mini Kit, as per the manufacturer's instructions (Qiagen, USA). One round of hybridization was then performed using TPA enrichment baits designed with the SureSelect protocol by Agilent (Agilent Technologies Canada, Inc., Mississauga, Ontario) (*1*). The samples then underwent Miseq sequencing, resulting in 300bp paired-end reads. The raw sequencing libraries quality controlled with fastp and filtered for reads originating from the *Treponema* genus using Kraken 2 (*2*,*3*). The filtered *Treponema* reads were mapped against the Nichols lineage (GCF_000410535) with BWA-MEM (H. Li, unpub. data, http://arxiv.org/abs/1303.3997). BCFtools was used to create consensus sequences from the mapped reads (*4*).

De novo Assembly of Public Library Sequences

827 raw sequencing libraries from two previously published papers were obtained from the Short Read Archives (SRA; *4*,*5*). These reads were similarly quality controlled with fastp and filtered using Kraken 2 (*2*,*3*). Following this, reads classified as originating from the *Treponema* genus were obtained used to create genome assemblies with Shovill (*6*). Assembly quality control was performed using both CheckM and Quast (*7*,*8*). Assemblies were excluded from the analysis if they contained $> 1\%$ contamination, $< 90\%$ marker gene completeness, or were not within 100kbp of the mean genome length of *T. pallidum*. Ten additional genomes were removed based on their phylogenetic positioning, likely due to genome mis-assembly. This led to a final count of 279 *T. pallidum* subsp. *pallidum*, three *T. pallidum* subsp. *pertenue*, and one *T. pallidum* subsp. *endemicum* genomes.

PopPUNK Lineages

Genomic lineages were called using a pre-existing POPulation Partioning Using Nucleotide Kmers (PopPUNK) database (*9*,*10*). This database was created using 827 *T. pallidum* genomes (544 assembled genomes from NCBI and 283 de novo assembled from previous papers) (*5*,*6*), across the three subspecies (784 TPA, 34 *T. pallidum* subsp. *pertenue*, and 9 *T. pallidum* subsp. *endemicum*). To minimize the effects of hypervariable regions on the clustering, the *tpr* gene family, along with *arp*, and *tp0470* were hard-masked (*12*). The *T. pallidum* PopPUNK database was created using a two-step process. The first was to create genomic

sketches of the 544 genomes from NCBI to serve as the initial groundwork for the database. During quality control, the SS14 lineage (GCF_000017825) was selected as the reference isolate, leading to the removal of 347 genomes. The remaining strains were then used to calculate nearest neighbor clusters using the PopPUNK lineage model. Following this, the 283 de novo assembled genomes as well as the 347 failed NCBI sequences were added to the PopPUNK database. *T. pallidum* lineages were defined using results from Rank 5 (*i.e.* clusters created using the five nearest neighbors of a strain). The Canadian strains not initially included in the PopPUNK database were typed during this study.

Confirming the lineages of the two Nichols strains

Upon the identification of two Nichols (CDN15 and CDN9) strains using the *T. pallidum* PopPUNK database (*13*), further confirmatory tests were performed. The first of these tests was to confirm the PopPUNK identification using a previously published three loci MLST (*14*). The three loci MLST confirmed that CDN15 was a member of the Nichols lineage (ST = 26, allele profile = 9.7.3). CDN9 failed during typing as the *TP0705* could not be identified, however, it shares the same first two alleles as CDN15, albeit with two SNPs in *TP0548* (allele profile = 9.7*.X).

The next step was to determine whether CDN15 and CDN9 were classified as Nichols due to an increased fraction of ambiguous nucleotides in the consensus genomes. This was not the case as both Nichols strains consisted of $\leq$ 1% Ns in their genomes (Appendix Figure 1).

The classification was finally confirmed using both a *k*-mer based dendrogram and a recombination masked core SNP phylogeny. Prior to both clustering and making the core SNP phylogeny, the *tpr* gene family, along with *arp* (*tp0433*), and *tp0470* were hard-masked to minimize the impact of these hypervariable regions (*12*). This was done by using coding sequences from EMBL-EBI and using BlastN (*15*) to identify their locations in the Canadian consensus sequences. BEDtools (*16*) was then used to mask the top blast hit for each gene in the genomes.

A subset of TPA genomes from GenBank from a previous publication (*10*) were chosen to act as the background when classifying the Canadian consensus genomes. The *k*-mer

dendrogram was created using Mashtree (*17*) with the genome size set to 1.2 Mbp. The resulting dendrogram was mid-point rooted.

A core SNP maximum likelihood (ML) phylogeny was constructed following previously published methods (*18*), using the TPA SS14 reference genome, GCF_000017825. In brief, a core SNP alignment was created using Snippy with additional recombination masking performed using Gubbins v3.3.5 (*19*,*20*). The resulting alignment was then filtered to retain only canonical nucleotides using SNP-sites (*21*). A ML phylogeny was then reconstructed with IQ-TREE 2 (*22*) with 1000 ultrafast bootstrap replicates. The best-fitting nucleotide substitution model was selected using ModelFinder (*23*) with an ascertainment bias correction.

The resulting dendrogram and core SNP phylogeny both firmly placed CDN15 and CDN9 within the Nichols lineage (Appendix Figures 2, 3, and 4).

16S and 23S rRNA Variant Calling

The 17 Canadian consensus genomes and 784 TPA genomes from the PopPUNK database were fragmented into 250 bp reads along a 5 bp sliding window using SeqKit and mapped against the Nichols 16S rRNA reference (NC_021490.2:231255–232803) using BWA-MEM (*24*; H. Li, unpub. data, http://arxiv.org/abs/1303.3997). Reads with a minimum mapping quality score of 30 were retained and deduplicated using SAMtools (*4*). Variant calling was performed using BCFtools (*4*), assuming diploidy due to the presence of two 16S and 23S rRNA genes in *T. pallidum*. The resulting VCF files were filtered for variants with a minimum quality score of 100 and were then subsequently normalized and merged into a single file.

Following the identification of nine genomes with a heterozygous T/G SNP at position 968 of the 16S rRNA gene, we performed a more stringent reanalysis using the raw reads of the sequencing libraries. More specifically, the sequencing libraries were filtered to retain only those classified as originating from *T. pallidum* prior to 16S rRNA mapping. This reanalysis did not detect the heterozygous variant, indicating that these SNPs were most likely the result of contamination from other *Treponema* species or sequencing artefacts.

References

1. Singh N, Braukmann TWA, Neale M, Long GS, Stein D, Van Caeseele P, et al. Complete genome sequences of two *Treponema pallidum* subsp. *pallidum* specimens from Canadian patients. Microbiol Resour Announc. 2025;14:e0064125. **PMID 40965152**

2. Chen S, Zhou Y, Chen Y, Gu J. fastp: an ultra-fast all-in-one FASTQ preprocessor. Bioinformatics. 2018;34:i884–90. PubMed https://doi.org/10.1093/bioinformatics/bty560

3. Wood DE, Lu J, Langmead B. Improved metagenomic analysis with Kraken 2. Genome Biol. 2019;20:257. PubMed https://doi.org/10.1186/s13059-019-1891-0

4. Danecek P, Bonfield JK, Liddle J, Marshall J, Ohan V, Pollard MO, et al. Twelve years of SAMtools and BCFtools. Gigascience. 2021;10:giab008. PubMed https://doi.org/10.1093/gigascience/giab008

5. Beale MA, Marks M, Cole MJ, Lee MK, Pitt R, Ruis C, et al. Global phylogeny of *Treponema pallidum* lineages reveals recent expansion and spread of contemporary syphilis. Nat Microbiol. 2021;6:1549–60. PubMed https://doi.org/10.1038/s41564-021-01000-z

6. Seemann T. tseemann/shovill [cited 2025 Jun 23]. https://github.com/tseemann/shovill

7. Mikheenko A, Prjibelski A, Saveliev V, Antipov D, Gurevich A. Versatile genome assembly evaluation with QUAST-LG. Bioinformatics. 2018;34:i142–50. PubMed https://doi.org/10.1093/bioinformatics/bty266

8. Parks DH, Imelfort M, Skennerton CT, Hugenholtz P, Tyson GW. CheckM: assessing the quality of microbial genomes recovered from isolates, single cells, and metagenomes. Genome Res. 2015;25:1043–55. PubMed https://doi.org/10.1101/gr.186072.114

9. Lees JA, Harris SR, Tonkin-Hill G, Gladstone RA, Lo SW, Weiser JN, et al. Fast and flexible bacterial genomic epidemiology with PopPUNK. Genome Res. 2019;29:304–16. PubMed https://doi.org/10.1101/gr.241455.118

10. Long G, Singh N, Patel S, Braukmann T, Tsang RSW, Duvvuri VR. Integrated genomic approaches improve *Treponema pallidum* phylogenetics and lineage classification. Can J Microbiol. 2025;71:1–11. PubMed https://doi.org/10.1139/cjm-2025-0021

11. Beale MA, Thorn L, Cole MJ, Pitt R, Charles H, Ewens M, et al. Genomic epidemiology of syphilis in England: a population-based study. Lancet Microbe. 2023;4:e770–80. PubMed https://doi.org/10.1016/S2666-5247(23)00154-4

12. Lieberman NAP, Lin MJ, Xie H, Shrestha L, Nguyen T, Huang ML, et al. *Treponema pallidum* genome sequencing from six continents reveals variability in vaccine candidate genes and dominance of Nichols clade strains in Madagascar. PLoS Negl Trop Dis. 2021;15:e0010063. **PMID 34936652**

13. Long GS, Singh N, Patel SN, Braukmann T, Tsang RSW, Duvvuri VR. Phylodynamics-and-AI-for-Public-health/TpallidumLineages [cited 2025 Jun 10]. https://github.com/Phylodynamics-and-AI-for-Public-health/TpallidumLineages

14. Grillová L, Bawa T, Mikalová L, Gayet-Ageron A, Nieselt K, Strouhal M, et al. Molecular characterization of *Treponema pallidum* subsp. *pallidum* in Switzerland and France with a new multilocus sequence typing scheme. PLoS ONE. 2018;13:e0200773. **PMID 30059541**

15. Camacho C, Coulouris G, Avagyan V, Ma N, Papadopoulos J, Bealer K, et al. BLAST+: architecture and applications. BMC Bioinformatics. 2009;10:421. **PMID 20003500**

16. Quinlan AR, Hall IM. BEDTools: a flexible suite of utilities for comparing genomic features. Bioinformatics. 2010;26:841–2. PubMed https://doi.org/10.1093/bioinformatics/btq033

17. Katz LS, Griswold T, Morrison SS, Caravas JA, Zhang S, den Bakker HC, et al. Mashtree: a rapid comparison of whole genome sequence files. J Open Source Softw. 2019;4:1762. PubMed https://doi.org/10.21105/joss.01762

18. Long GS, Klunk J, Duggan AT, Tapson M, Giuffra V, Gazzè L, et al. A 16th century *Escherichia coli* draft genome associated with an opportunistic bile infection. Commun Biol. 2022;5:599. PubMed https://doi.org/10.1038/s42003-022-03527-1

19. Seemann T. tseemann/snippy [cited 2025 Jun 27]. https://github.com/tseemann/snippy

20. Croucher NJ, Page AJ, Connor TR, Delaney AJ, Keane JA, Bentley SD, et al. Rapid phylogenetic analysis of large samples of recombinant bacterial whole genome sequences using Gubbins. Nucleic Acids Res. 2015;43:e15. PubMed https://doi.org/10.1093/nar/gku1196

21. Page AJ, Taylor B, Delaney AJ, Soares J, Seemann T, Keane JA, et al. SNP-sites: rapid efficient extraction of SNPs from multi-FASTA alignments. 2016;2:e000056. **PMID 28348851**

22. Minh BQ, Schmidt HA, Chernomor O, Schrempf D, Woodhams MD, von Haeseler A, et al. IQ-TREE 2: New Models and Efficient Methods for Phylogenetic Inference in the Genomic Era. Mol Biol Evol. 2020;37:1530–4. PubMed https://doi.org/10.1093/molbev/msaa015

23. Kalyaanamoorthy S, Minh BQ, Wong TK, Von Haeseler A, Jermiin LS. ModelFinder: fast model selection for accurate phylogenetic estimates. Nat Methods. 2017;14:587–9. **PMID 28481363**

24. Shen W, Le S, Li Y, Hu F. SeqKit: a cross-platform and ultrafast toolkit for FASTA/Q file manipulation. PLoS One. 2016;11:e0163962. PubMed https://doi.org/10.1371/journal.pone.0163962

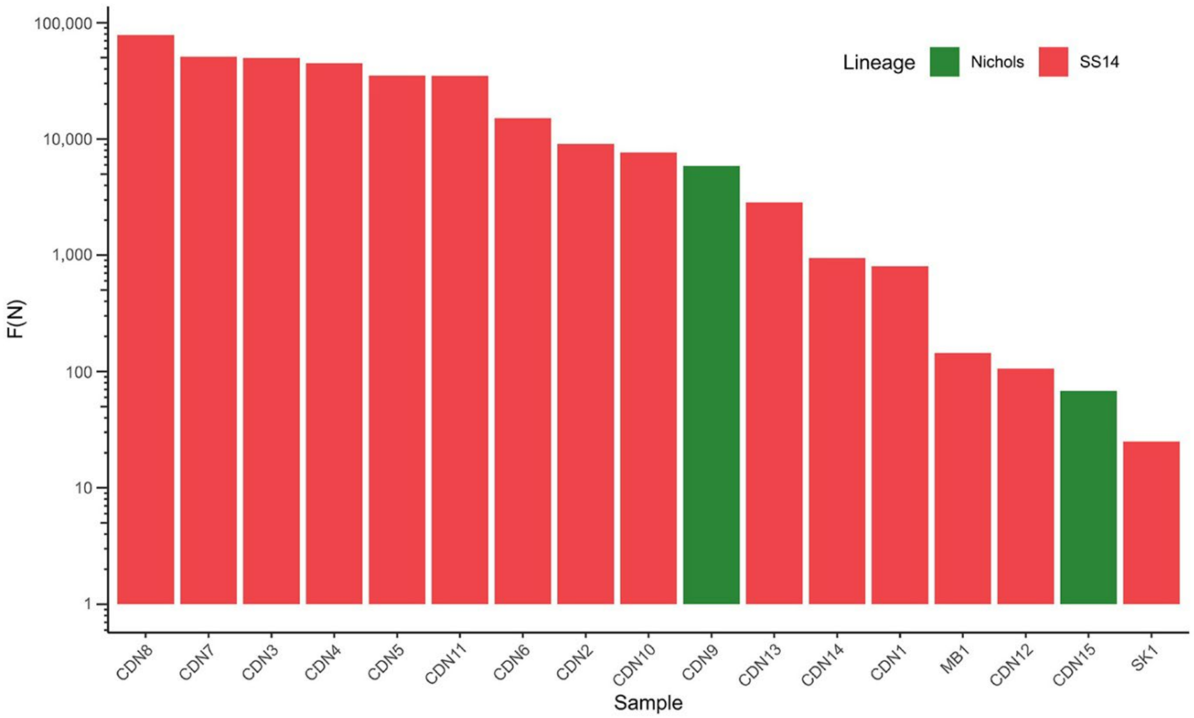

Appendix Figure 1. Number of ambiguous nucleotides in the Canadian consensus genomes.

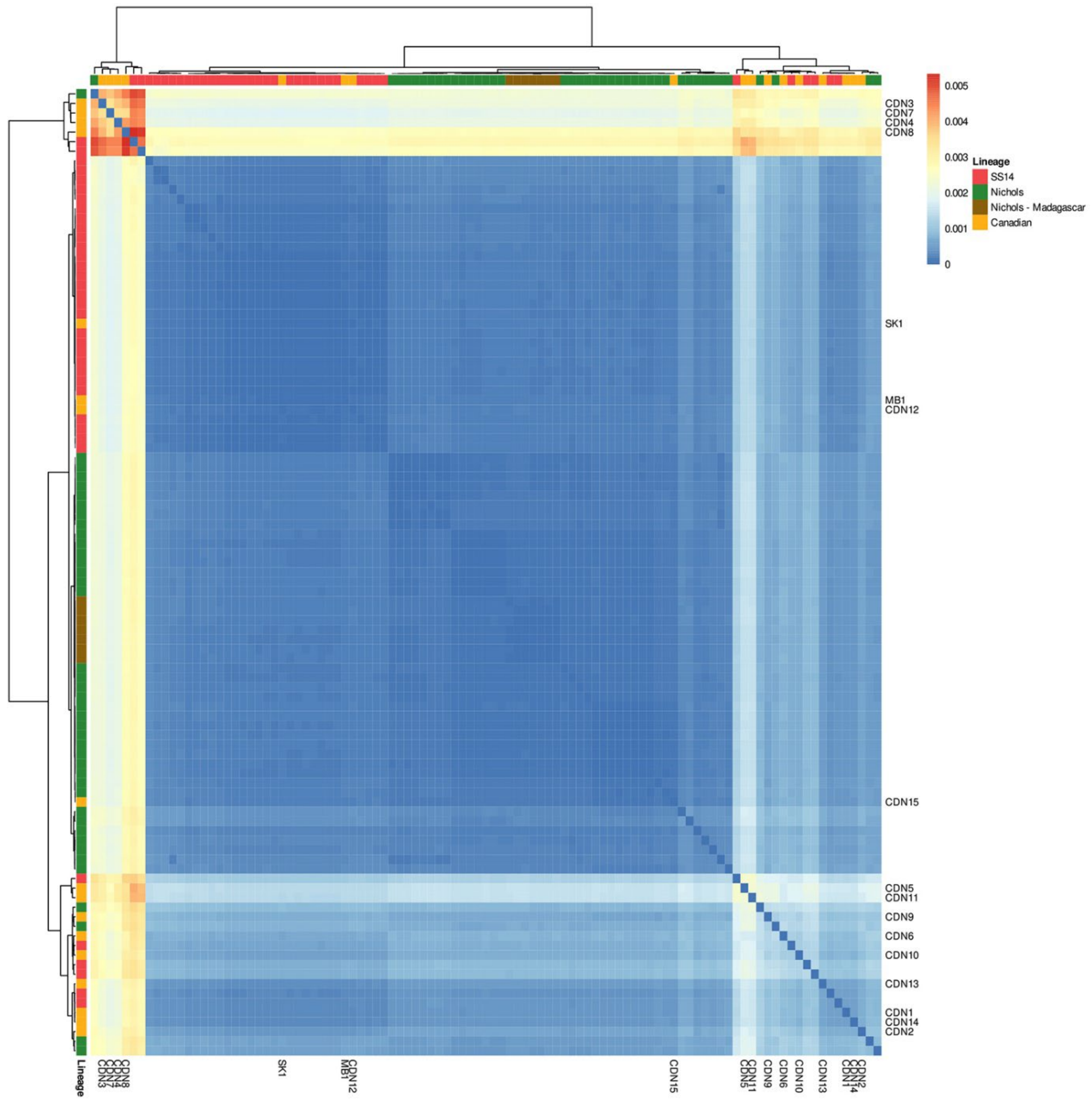

Appendix Figure 2. *K*-mer distance heatmap of the Canadian *T. pallidum* consensus sequences and GenBank *T. pallidum* subsp. *pallidum* genomes. Distances were calculated using MashTree (*17*). Labels indicate Canadian genomes.

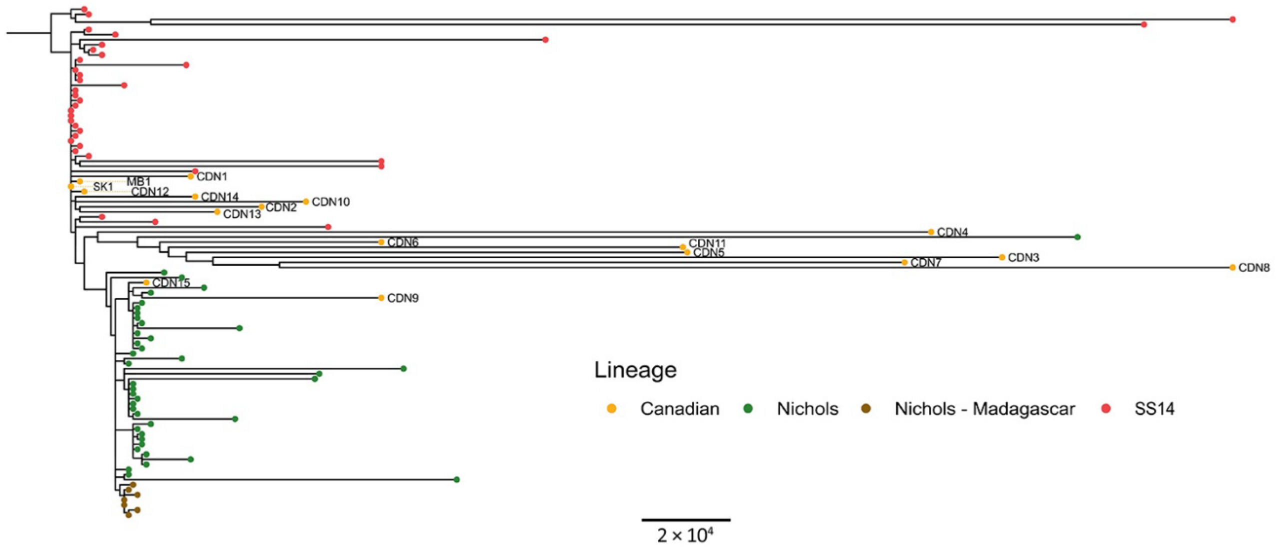

Appendix Figure 3. *K*-mer dendrogram of *Treponema pallidum* subsp. *pallidum*. A *k*-mer based dendrogram created using Mashtree (*17*) showing the relative similarities of the 17 Canadian genomes within TPA.

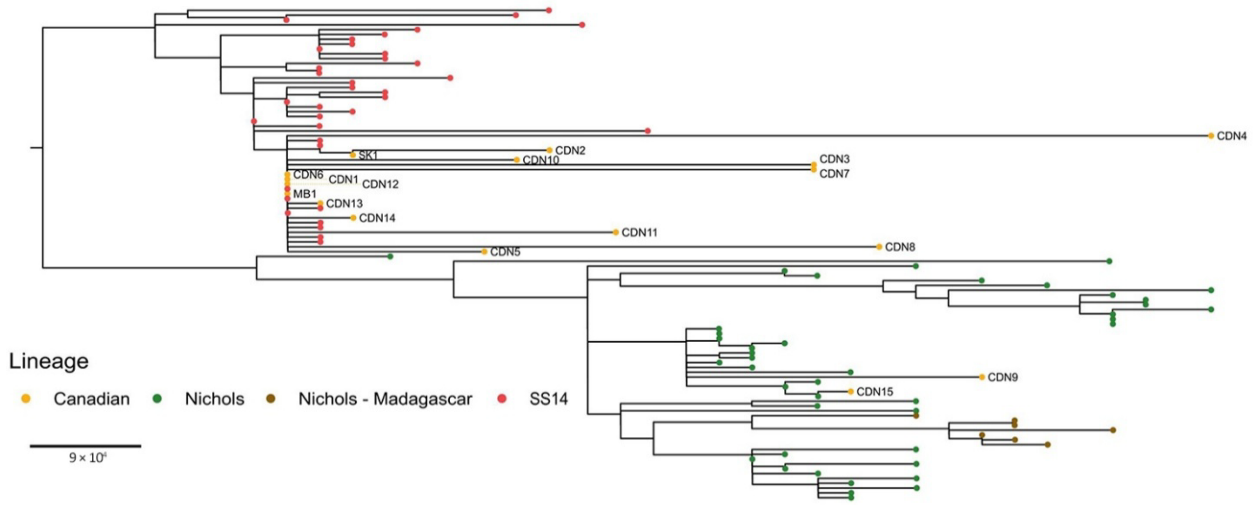

Appendix Figure 4. Core SNP phylogenetic tree of *Treponema pallidum* subsp. *pallidum*. A core SNP phylogenetic tree of TPA.
